# Extra Supplement—The Nursing Mirror

**Published:** 1890-09-27

**Authors:** 


Being the Extra Nursing Supplement of "The Hospital" Newspaper.

All Contributions for this Supplement should be addressed to the Editor, The Hospital, 140, Strand, London, W.C., and should have the word "Nursing" plainly written in left-hand top corner of the envelope.

### En Passant.

Dartmouth Cottage Hospital.—A very successful fancy ball took place at Dartmouth on the 2nd inst., in aid of the funds of the Cottage Hospital. It was held in the subscription-rooms, which were gay with flags, flowers, and Japanese lanterns. This hospital is almost entirely supported voluntarily, and we are glad to hear that the Committee cleared £45 by the aid of these festivities.

New Medical School for Women.—October 21st will see the opening of the Medical School for Women, in connection with Queen Margaret College, Glasgow. The regulations, fees, and curriculum will be similar to those at Glasgow University. The hospitals are the Glasgow Maternity and the Royal Infirmary, at which last wards are to be reserved exclusively for the use of the students of the college. The Hon. Secretary is Miss Galloway, Queen Margaret College, Glasgow.

Notes and Queries.—Of the last-mentioned we receive a good many; and we are glad to have them. We should be pleased to receive a great many more answers than we do. Many people never send an answer to a newspaper query because they think there will be so many answering at the same time, but if everybody thought that the result would be sad. Supposing somebody asks a question about work abroad, the chances are that amongst our numerous readers somebody has had personal experience of the work the inquirer wants information about, which would be invaluable. So the sender of the "Note" would earn the gratitude of Mr. Editor and the inquirer as well.

The Rural Nursing Association, formed for supplying nurses thoroughly trained as midwives to the rural poor, issued last week a strong appeal to the public for funds. Sir John Lubbock has kindly consented to act as one of the treasurers, and Mr. G. E. Martin, of Worcester, as the other. Ultimately the association hopes to form branches in each county in England, in order to carry out the work effectually. This is an admirable undertaking; still the great hope lies in the time when the law will insist that none but registered midwives shall attend women in their confinements. Now, perhaps, one of the most difficult things to accomplish is to make the poor really grasp the fact that there is something advantageous in having a trained nurse. If they can once be persuaded to try what comfort is derived from superior nursing, they will not only want no other, but will persuade others to appreciate it. We have so often heard country women say, when we have asked if they have a woman coming in during the time they are laid up, "Well, I have Mrs. So-and-so coming in, she's a useful body, and do for me nicely, and sees to the washing," and very often Mrs. So-and-so does "do" nicely, sometimes fatally for the poor women. Ladies who have friends among the poor can do as much to pave the way for trained nurses as anybody by pointing out the advantages of having them. A great deal of it is mere prejudice. We know of a village where the new district nurse was not at all appreciated at first, and where now she is invaluable. One or two women were nursed by her and the others followed like a flock of sheep.

In Russia, a recent notice permits female practitioners to be appointed district medical officers, with charge of the district hospitals.

The Dufferin Fund.—The Central Committee of the Dufferin Fund at Simla has decided to form three new scholarships to be held at the principal colleges in India. This resolution is the result of the anxiety of numerous ladies to become doctors.

Miss Kate Marsden, who has been permitted by the Empress of Russia to visit various parts of Russia and Siberia, for the purpose of seeing and noting the condition and method of treatment of the lepers in that country, left on Friday last, the 19th inst., by the mail steamer *Parramatta*.

Hints for Christmas.—A correspondent sends us a suggestion for providing some of the numerous treasures which are being collected for the children at Christmas time. Half sheets of letter paper, which are usually saved by the thrifty and condemned to the paper-basket by the thriftless, if collected and made up into little books with a pencil attached to them by a string, would make admirable drawing books. Coloured scraps can be had for a penny a sheet, and if one scrap—pictures of birds, animals, flowers, &c.—were stuck at the top of each leaf of these books, they would afford children pleasure for days together copying them. We may suggest that if the last leaf were made of cardboard it would be still better, for then the small occupants of cots, if allowed to sit up, would have something substantial to scribble upon.

"Our Duty is to make ourselves useful, and thus life may be most interesting and yet comparatively free from anxiety," says Sir John Lubbock, and he certainly seems to find his life interesting judging by the amount of ways in which he makes himself useful. It is ever so much more comforting to hear good advice from a man whose life shows that he preaches what he practises than to read the best theoretical sermon in the world. It's the lives of really great men all around us which are such a help when we make an effort and there seems very little result from it, for we know that success did not come and crown their efforts at the very beginning. Just to think of the lives of those in the nursing world. Nobody who knows anything about it would say that nurses have nothing in the shape of disagreeables and difficulties to meet with, but they will certainly say that a nurse's life can be "most interesting," that is to say if she goes about her work thoroughly, throwing all her energy and intelligence into it. Duty can be an immense happiness; it is the achieving of something which makes happiness. There are so many who find life flat and stale, who if they went in for some sort of duty thoroughly would feel quite different. For instance, if nurses looked upon their career as merely a means to bread and butter, if they were upset by every trivial misunderstanding their lives would not be a bit useful, and they themselves would have the horrid consciousness that there was no "interest" in their lives, and there would certainly be no result. Enthusiasm is often ridiculed, but still really enthusiastic people have a great deal of happiness. To strain ourselves to excel in whatever we attempt in life means infinite contentment, and we may just as well be happy as miserable.

### Sketches of Insanity.

By Francis H. Walmsley, M.D. (Senior Assistant Medical Officer of Leavesden Asylum).

### XII.—PUERPERAL INSANITY.

Puerperal mania is a very distressing malady in itself, but doubly so from occurring at a moment ordinarily so joyful.

Patients are generally attacked during the period between the fourth and the fifteenth day, though they are specially susceptible about the fourth or fifth day after confinement. In many cases the onset is sudden, the patient waking up from what appeared to be a healthy slumber; or the disorder may be ushered in by a degree of exhaustion, conjoined with great excitability, headache, and want of sleep; or again, the attack may accompany or follow convulsions. The face is usually pale and haggard, with occasional flushing, the expression is indicative of alarm and suspicion, the eyes assume a morbid lustre—are unnaturally brilliant, and wildly glance at objects in rapid succession—the skin is often dry sallow or jaundiced. The patient may be joyous or melancholy, singing and talking incessantly, or silent, and suspicious of every one about her, fancying injuries and offences on the part of her husband or friends, and forgetful of her child.

The ideas are at first rapid and confused and of the most extravagant kind, images like those of dreams appear, and the delirium is soon confirmed by these illusions being considered as realities, the speech and actions corresponding with these impressions. The dominant mood is one of suspicion and distrust; the patient manifests strong antipathies, is dissatisfied with those in attendance on her, and imagines they are in league to do her some grievous harm; she takes a violent and bitter aversion to her husband and child; she is restless and agitated, struggles desperately, makes persistent efforts to jump out of bed, and is not in the least affected by any exhibition of restraining power; the sleep is fitful, and disturbed by dreadful dreams. Again, the patient may be in a state of frenzy—beside herself—wild with terror, and apprehensive of some impending calamity.

There is developed a condition of extreme sensitiveness, with great intolerance of light and of the slightest sound; the sense of taste is very often perverted, and thus there arises in the patient's mind the belief that her food is poisoned, she cannot be induced to eat, therefore it becomes necessary to resort to forcible feeding. There are frequent explosive outbursts of passion with tendency to sudden impulsive acts of violence; the patient may attempt to destroy herself, her child, or persons for whom she has usually the most affectionate regard; she apparently hears voices prompting her to strangle herself or to throw herself out of the window; believes she is surrounded by devils, evil spirits, witches, from whom she makes frantic efforts to escape; she listens to and shouts at imaginary persons whose faces she fancies she sees peering in at the window. A disposition to murder the offspring is a most prominent symptom; there is apparently an entire want of power to control the murderous feeling; there is no attempt at concealment, nor any denial of the crime on detection; conscious of this uncontrollable impulse, the unhappy mother, at times, begs that the child may be taken from her.

The number of cases that recover is very considerable—as many as eighty out of every hundred, the great proportion of recoveries taking place during the first three or six months; a fatal termination need not be anticipated in more than five per cent. of the cases. Certain patients are attacked after the birth of each child, becoming more or less permanently affected after the third or fourth attack.

In many cases—in half the number, or possibly more—hereditary predisposition is a potent factor in the causation of this distressing malady.

(*To be continued.*)

### A Catholic Nurse's Holiday.

The chief trouble of a district nurse is that she can do so little compared with what is required. Many lives are saved, many more comforted and cheered by the daily or more frequent visits of a trained nurse. Wounds are dressed regularly and without unnecessary pain, patients are kept clean (or at least *made* clean), poultices are put on hot, typhoids are *not* sat up in a chair to have their beds made, bedsores are prevented, and the sense of being thought of and cared for softens the hard lot of many a sick man and woman. Those who provide these "servants of the poor," do very much to alleviate suffering, and there is a wide field for the exercise of love and devotion in the work of a district nurse. Still there is the trouble. Let me recount a few instances.

(1). An old woman lies dying of heart disease in one corner; her daughter, the mother of five children under seven lies moaning with pain in a bed in the same room. The old woman keeps lingering on, and the daughter cannot make up her mind to hasten the final parting by being moved to a hospital. Morning and night the district nurse comes, washes patients, makes beds, and does such odds and ends of nursing and tidying as come to hand. During her early morning visits the father, grimy and tired after a night's work in the mine, washes and dresses and starts off to school the four middle babies—a proceeding often interrupted by a sounding kiss and a fond "God love thee!" The eldest stays at home to help, and to mind the youngest, an infant. At night the man is often obliged to delay going to his work, because no one has come to take care of the invalids and children, or to fetch medicine, &c., after they have come. After the old mother is dead, the wife just about, and the poverty more urgent than usual, the man is dismissed for non-attendance. Imagine the feeling of a nurse obliged day after day to leave so much misery scarcely touched.

(2). A man has cut his throat and then falling, broken his leg, without displacing the bone. Morning and night the doctor feeds him with liquids poured through a long tube into his stomach, and even his skill and care cannot prevent fits of choking sending food and phlegm up through the gaping wound. Every movement is agony to the broken thigh or the throat, the poor mouth is parched, the voice a hollow faint murmur. There he lies, faint, thirsty, tormented with pain, with death or imprisonment before him. Here is constant work day and night for skiful tender hands! The steam must be kept just right, the tent also. A pillow shifted and changed while the head is held steadily so that that terrible wound is not widened or irritated by coughing. The sponges on the throat must be washed and changed frequently and the parched mouth moistened and washed out. The position must be shifted, the linen straightened and changed so gently, and a thousand and one little things done to ease the physical and mental agony of such a case. Be it insanity, drink, or despair that has brought this about what does it matter? The nurse sees what is only, and Oh! it is hard to leave such a case most of the day and night to relatives who curse him and only do what they must, and to kindly but uncertain neighbours!

(3). A woman who has first had her head cut open and then got badly burned over her head and shoulders. She has no friend in the place except the man who has first caused all this, and then kept her hidden for days without doctor or nurses. Now, with nerves shattered and in terrible pain, she is left for the greater part of the day and night at the mercy of this brute. Half-crazed with the pain and shock, with deep sloughing wounds and immediate danger of blood-poisoning, she cannot be moved the seven miles to the nearest hospital, and day after day the nurse leaves her, fearing for her reason, if not for her life.

These are only a few typical cases. Of the typhoids who fall out of bed, run about in delirium, or are found with beef or oatmeal cakes or crumpets; of the bronchial cases found with one tiny cool poultice on the stomach; of the babies dying because they are too weak to suck, and no one feeds them; of the patients sinking because ignorant attendants will not force them to take food frequently, there is not time to speak. Only those whose daily work lies amongst the sick poor know how much the district nurse is obliged to leave undone.

So much as to the demand; now a few words as to the supply.

Twenty-five years ago a few Catholic women, with the assistance of a priest with whom the plan originated, began, in poverty and difficulty, the work of nursing the sick poor in their homes. This was in Paris. To-day there are seventeen houses in France and two in England, and they are asked for in America, Africa, Ireland, and the Canary Isles! The idea is to be as the mother or sister or daughter in the cottage, giving all, taking nothing, setting any member of the family who can earn money free to earn it, shopping, cooking, sending children off to school, minding the baby and the sick person; in fact, doing what there is to do, keeping everything going, and so averting the worst evils of sickness amongst the very poor. To be apostles, to raise the spiritual and moral condition of those they work amongst, to reach the soul through the body, is the primary aim of the order; but the sick poor of all denominations are attended, and no religion held in good faith is interfered with. It is also a rule to carry out loyally all doctor's orders, and, if necessary, a night nurse is provided also.

To the mother house of this community, the "Little Sisters of the Assumption, 57, Rue Violet, Grenelle, Paris," I went for my summer holiday this year. What a lovely run it is, in June, from our dear, dirty, smoky districts, out into the beautiful bold, north country scenery of hill and dale, and rapid brown streams, through the peaceful meadow lands and farms, the yellow fields, and fir plantations of the midland counties. A glimpse of busy London and old hospital chums, and then the grassy hills and flowery fields and hedgerows of the south, till we reach the sea at last, and seem to feel ourselves growing stronger with each breath of the salt fresh air. By starlight, moonlight, or sunlight, the passage across the Channel is delightful, but Dieppe looks especially charming in the grey light of dawn, and the pasture lands, green hills, rivers, quaint farmhouses, and the lovely foliage of the trees on the way to Paris gain additional charms from the glory of a rising sun. And Paris itself at eight o'clock on a June morning has quite a refreshing air of sweet simplicity!

Grenelle is an unfashionable quarter of Paris. The convent has a large garden, walled in, and so quiet and shady, and sweet with the scent of flowers, that it is rest and enjoyment for a nurse, tired of ugliness and noise, just to sit there, dreaming or reading, listening to the wind amongst the tall trees, or watching the blue sky through the waving boughs. Such lazy enjoyments are for holidaymakers only, however; the community is as busy as a beehive, but without the buzz. The founder lives not far off, and is often at the convent; the foundress is dead, and there is a second Mother-General. About 150 professed sisters, novices, and postulants live at the Mother House, or, rather, in the grounds, for the first-year novices have a house to themselves, where with prayer, and study, and holy discipline they prepare for the fight with the misery and sin in the world. From England, Ireland, Spain, Germany, Russia, and all parts of France; simple, gentle, and noble; leaving fortune and luxury, or dear humble homes, or friends dearer than life; bringing culture, and talents, and learning, or simple household knowledge, girls who have turned away from pleasure at the first sight of it, and women who, knowing the pains and joys of the world, have chosen the better part, they come to this convent, and after a few weeks' retirement and quiet meditation exchange picturesque cap or fashionable hat for the postulant's cap, and so enter on the months of trial before they take the habit of a novice. There are no servants or lay sisters, so there is plenty of work to fill the spare time of novices and postulants. There are sisters to overlook and portion out the work of the mission, and the young novices, who have generally had no previous training, are instructed by experienced sisters in all branches of the work, and are not sent to a case they are not competent to undertake. Health and a vocation are the two things indispensable for anyone aspiring to be a Little Sister of the Assumption. As the visitor of my sister, a trained hospital and private nurse, who has joined the order, I saw and heard much of the inner life of the community, and was particularly struck with its spirit of simplicity, order, and thoroughness. The discipline is strict, the poverty real, but there is plenty of spirit and "go" in this convent, and those nurses who contemplate entering religion will do well to think of this, primarily, nursing order. I may add that there is more of the contemplative life also here than in any other active community.

A glimpse at Brighton; a day at Ventnor, perfectly beautiful in her fresh summer foliage, with her shaded green and blue and purple sea, steep wooded hills, broken cliffs, and luxuriance of wild flowers. Another glimpse at London, a run home by another line, and my district again!

FRANCISCUS.

### Six Months in a Hospital.

(*By a "Special Pro."*)

MY DEAR CASSY,—This is to inform you that, much to my own surprise, although by my own deed, the die is cast, my ships are burnt, and at the end of this month I cross the Rubicon; otherwise the threshold of the B—— Hospital. I came from Bournemouth on Friday, and found a letter awaiting me, requesting a prompt interview with the matron, who was willing to take me in. Next morning off I went; and in utter disgust at my want of resolution settled the matter.

These are my terms of entrance. I go as a "special," and engage for six months only, after which time I am entirely free. I attend all lectures, etc., given, go through a medical and surgical course, and as a "special" I go on duty one and a-half hours after the other nurses, by which time Miss S—— says all the disagreeably hard work is done. I also have my bed made, boots cleaned, hot water brought up, and various little matters of that kind done for me which the other nurses have to do for themselves, and two hours off every day. Now I think this would just suit you. Brace up your mental and moral mind, and ask your mother to let you come for six months. I am (excepting one girl, who actually engaged herself first) the first "special" seen within the walls of the B—— Hospital. The matron is charming, so pretty, and young. She seemed quite pleased to have me, and said she was sure I should make a very good nurse. She says that six months' training will teach you all you want for sick nursing at home amongst friends; although, of course, certificates are never given under a three years' training. One other advantage I have, and that a great one. If at the end of six months I am suitable and want to stay on, I shall have the first vacancy that occurs in the staff nurses offered to me, and can then enter the regular course.

I go to-morrow to get my uniform, bonnet, and long cloak, striped blue and white print, collars and cuffs, and the usual becoming big apron. The caps are not bad, but tie under the chin, which I do not like. I go at eight o'clock on March 1st. Think of me! I believe if you were coming too, my joy would be perfect. I have no scrubbing, no fire-place "sides," nothing in kitchen, and am not to be sent on errands about the hospital, *i.e.*, fetching bottles, etc. I have dusting, washing up mugs, and may have perhaps a little sweeping; possibly none. The nursing work will be, of course, similar to that of the others.

I cannot tell you how much happier I have felt since this matter has been settled. Life has suddenly taken on a definite and tangible shape for me; it seems no longer made up of fragments and shreds which the first wind scatters again after all our trouble in heaping them together. I seem to have found a foothold in what has been before only a shifting sand, and Heaven only knows what the misery of living on that shifting sand is!

(*To be continued.*)

### Appointments.

[It is requested that successful candidates will send a copy of their applications and testimonials, with date of election, to The Editor, The Lodge, Porchester Square, W.]

Miss M. Agnes Wright has been appointed matron of the Convalescent Hospital, Cheadle, near Manchester, and took charge of it on the 6th inst. Miss Wright was trained at the Derbyshire General Infirmary, where she afterwards held wards, and then went as home sister to the Manchester Royal Infirmary till she obtained this present appointment.

Kimberley.—Miss Rose Blennerhassett, trained at the Salop Infirmary, Lambeth General Lying-in (Obstetric Society's diploma), superintendent-nurse Cardiff Union Hospital, district nurse Johannesburg Nurses' Home, to be night superintendent at Kimberley Hospital (300 beds), Cape Colony.

Stratford-on-Avon.—Miss Adelaide J. Lloyd, late sister at the Seamen's Hospital, Greenwich, has been appointed matron of the Stratford-on-Avon Hospital.

### Notes and Queries.

To Correspondents.—1. Questions may be written on post-cards. 2. Advertisements in disguise are inadmissible. 3. In answering a query please quote the number. 4. If a private answer is desired, a stamped addressed envelope must be forwarded.

Notice.—We must really ask our correspondents to be more particular in enclosing name and address. Constantly we receive queries or letters with no name attached.

### Queries.

(38) *Rex* would be glad to know where she can learn dispensing, preferably in or near Liverpool. She would like to attend as a non-resident pupil, paying not more than £5 5s. for the course, but she would give extra time if required.

(39) *Nurse* will be glad if some one will tell her whether Bombay is a good place for English nurses to obtain posts, also the names of the hospitals there, and any other information.

(40) *H. C. H.* would be glad if any matron can tell her in next week's "Hospital" where patient's linen sheeting, with two narrow stripes (red or blue), may be obtained, and price.

### Answers.

(36) *Nursing Abroad.*—Many nurses have gone to South Africa, to the Kimberley and Port Elizabeth Hospitals. At Johannesburg a hospital and nursing institution is being started by Miss Mollett (late matron of Chelsea Infirmary). For India, Lady Dufferin's Fund sends out nurses, and the address of the secretary is Colonel Robertson, 65, Kensington Gardens Square, London. Lady Roberts's Fund sends out nurses; apply to Major Cooper, 4, Upper Phillimore Gardens, London. The Cyprus Society are shortly going to send nurses to the island; apply 30a, Wimpole Street. *Nursing Abroad* had better advertise saying what particular spot she wishes to go to.

(37) *Toxteth* had better apply to the chief London and provincial hospitals for their rules respecting probationers. Certificates are given at the end of three years at St. Bartholomew's and Guy's, or at Edinburgh Royal Infirmary. She had better inquire at these.

### Vacancies.

The following vacancies are announced:

### Assistant Superintendent.

General Infirmary, Leeds; £50.

### Nurses.

Atkinson Morley's Convalescent Hospital, Wimbledon; £18.

Bangor Institute.

Blackheath and Richmond Institutions.

Birkdale Home, Southport.

Borough Hospital, Birkenhead; £25.

Bournemouth Institution; £25.

Cambridge Home, Cambridge.

Cheltenham Institution.

Christ's Hospital, Hertford; £24.

City and County Institution, Worcester.

Frome Home; £22.

Hanover Institute, Hanover Square, W.; £25.

Leeds District Home; £25.

Levick Institution, Paris.

Mercer's Hospital, Dublin; £20.

Mr. Wilson's Institution, Wimpole Street, W.

Norwich Home.

Newark Hospital; £18.

N.R. Infirmary, Middlesbrough-on-Tees; £25.

Nurses' Institution, Erskine Street, Liverpool; £25.

Royal Hospital, Landport; £25.

Queen Victoria Institution, Wolverhampton.

Royal Infirmary, Newcastle-on-Tyne; £20.

Samaritan Free Hospital, N.W.

St. John's Institute, Southport; £27.

South-Eastern Fever Hospital, S.E.; £30.

South London District Association.

St. Leonard, Shoreditch; £17 and £20.

St. Asaph Union; £25.

Sheffield Home; £25.

Sydney Home; £30.

St. Helena Home, Grove End Road, N.W.

St. Saviour's Infirmary, S.E.; £20.

Victoria Cottage Hospital, Guernsey; £25.

Victoria Home, Bournemouth; £25.

Wirral Children's Hospital, Birkenhead.

Weston-super-Mare Institute; £20.

Western General Dispensary, N.W.; £25.

Workhouse Infirmary Association, Adam Street, W.C.

### Probationers.

Batley Hospital, Yorks.

Children's Hospital, Derby.

Leeds Institution.

Metropolitan Convalescent Institution, Kingston Hill.

Monsall Fever Hospital, Manchester; £15.

Oldham General Infirmary.

St. Helena Home, Grove End Road, N.W.

Taunton and Somerset Hospital.

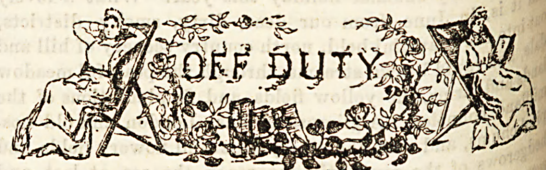

### Amusements and Relaxation.

**SECOND QUARTERLY WORD COMPETITION.**

Commenced July 5th, 1890, ends Sept. 27th, 1890.

Three prizes of 15s., 10s., 5s., will be given for the largest number of words derived from the words set for dissection.

Proper names, abbreviations, foreign words, words of less than four letters, and repetitions are barred; plurals, and past and present participles of verbs, are allowed. Nuttall's *Standard* dictionary only to be used.

N.B.—Word dissections must be sent in WEEKLY not later than the first post on Thursday to the *Prize Editor*, 140, Strand, W.C., arranged alphabetically, with correct total affixed.

The word for dissection for this, the THIRTEENTH week of the quarter, being

"CRICKHOWELL."

| Names. | Sept. 20th. | Totals. | Names. | Sept. 20th. | Totals. |
|---|---|---|---|---|---|
| Dulcamara | — | 503 | Jenny Wren | 21 | 603 |
| Tinie | 24 | 647 | Rhoda | — | 242 |
| Henri | — | 395 | Annabel | — | 17 |
| Agamemnon | 27 | [illegible] | Spare Moments | — | 19 |
| Patience | 24 | 647 | Qu'appelle | — | — |
| Brisbane | — | 332 | Nurse | — | 29 |
| Nurse Hilda | 22 | 622 | A. B. C. | — | 251 |
| Lightowlers | 28 | 645 | Brickbat | — | 77 |

N.B.—Third Quarterly Word Competition commences Oct. 4th, 1890, ends Dec. 27th.

### Notice to Correspondents.

N.B.—Each paper must be signed by the author with his or her real name and address. A *nom de plume* may be added if the writer does not desire to be referred to by us by his real name. In the case of all prize-winners, however, the real name and address will be published.

Competitors can enter for *all* quarterly competitions, but no competitor may take more than *one first prize* during the year.

